# A Perl procedure for protein identification by Peptide Mass Fingerprinting

**DOI:** 10.1186/1471-2105-10-S12-S11

**Published:** 2009-10-15

**Authors:** Alessandra Tiengo, Nicola Barbarini, Sonia Troiani, Luisa Rusconi, Paolo Magni

**Affiliations:** 1 Dipartimento di Informatica e Sistemistica, Università degli Studi di Pavia, Via Ferrata 1, I-27100 Pavia, Italy; 2 Biotechnology dept., Nerviano Medical Sciences, Via Pasteur 10, I-20014 Nerviano, Italy

## Abstract

**Background:**

One of the topics of major interest in proteomics is protein identification. Protein identification can be achieved by analyzing the mass spectrum of a protein sample through different approaches. One of them, called Peptide Mass Fingerprinting (PMF), combines mass spectrometry (MS) data with searching strategies in a suitable database of known protein to provide a list of candidate proteins ranked by a score. To this aim, several algorithms and software tools have been proposed. However, the scoring methods and mainly the statistical evaluation of the results can be significantly improved.

**Results:**

In this work, a Perl procedure for protein identification by PMF, called MsPI (Mass spectrometry Protein Identification), is presented. The implemented scoring methods were derived from the literature. MsPI implements a strategy to remove the contaminant masses present in the acquired spectra. Moreover, MsPI includes a statistical method to assign to each candidate protein, in addition to the scoring value, a p-value. Results obtained by MsPI on a dataset of 10 protein samples were compared with those achieved using two other software tools, i.e. Piums and Mascot. Piums implements one of the scoring methods available in MsPI, while Mascot is one of the most frequently used software tools in the protein identification field. MsPI scripts are available for downloading on the web site .

**Conclusion:**

The performances of MsPI seem to be better than those of Piums and Mascot. In fact, on the considered dataset, MsPI includes in its candidate proteins list, the "true" proteins nine times over ten, whereas Piums includes in its list the "true" proteins only four time over ten. Even if Mascot also correctly includes in the candidates list the "true" proteins nine times over ten, it provides longer candidate lists, therefore increasing the number of false positives when the molecular weight of the proteins in the sample is approximatively known (e.g. by the 1-D/2-D electrophoresis gel). Moreover, being MsPI a Perl tool, it can be easily extended and customized by the final users.

## Background

Protein identification is one of the hardest tasks in proteomics and over the years many high-throughput technologies and methods have been developed for improving it. Mass spectrometry has become a key tool for protein identification, being able to measure with high precision the mass/charge ratio (m/z) of charged molecules such as peptides. However, due to the large amount of generated data, protein identification represents from a computational point of view one of the major challenges in proteomics [1,2].

**Figure 1:**
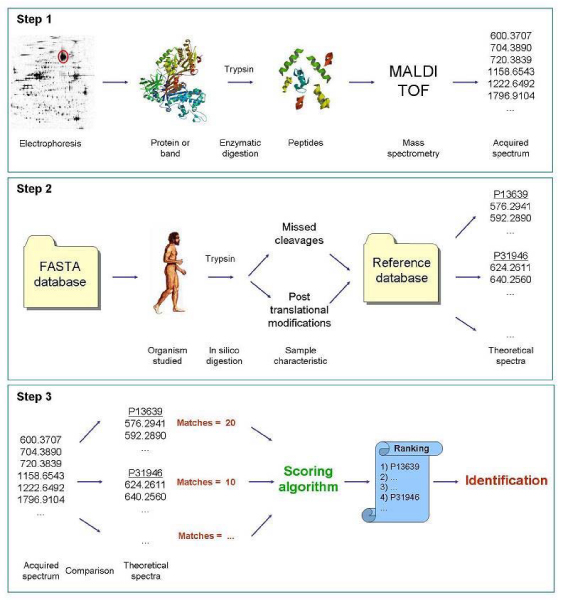
**PMF consists of three steps**. (1) The preparation of the biological sample: a band or a spot of the electrophoretic gel is selected and digested by a suitable protease, such as trypsin. The resulting mixture of peptides is analyzed with a mass spectrometer, usually in MALDI-TOF configuration. (2) A reference protein database is created, reproducing *in silico *on a set of known proteins the step 1, considering also possible missed cleavages and post-translational modifications. (3) The acquired spectrum is matched against the theoretical spectra generated by *in silico *digestion of all proteins in the reference database (step 2) and a ranked list of candidate proteins is obtained.

Protein identification by mass spectrometry can be performed following two approaches: the Peptide Mass Fingerprinting (PMF), relying on single-stage MS, and the Peptide Fragment Fingerprinting (PFF), which is based on tandem mass spectrometry [3]. This paper only copes with the former approach. PMF allows the identification of a protein by combining MS data with searching strategies on a suitable protein database, provided that the amino acid sequence of the protein in the biological sample is already known and stored in the database. Therefore *de novo *protein identification cannot be performed by PMF. As illustrated in Figure 1, the PMF workflow can be subdivided in the following three steps:

1. sample preparation and spectrum acquisition;

2. reference protein database generation;

3. matching of the acquired spectrum against the generated reference protein database.

### 1. sample preparation and spectrum acquisition

Proteins are separated from other cellular components and resolved by gel electrophoresis on the basis of either molecular weight (MW) only (1-D electrophoresis) or both isoelectric point (pI) and MW (2-D electrophoresis). Protein bands (or spots in 2-D) are excised from the gel and digested with a protease that cleaves the protein at specific peptide bonds depending on the amino-acid sequence, generating a pool of peptides [4]. The mixture of peptides obtained by enzymatic digestion is then analyzed by mass spectrometry, often in MALDI-TOF configuration, for obtaining the mass spectrum of the whole mixture.

### 2. reference protein database generation

The enzymatic digestion of all the known proteins of interest is reproduced *in silico*. The result of this task is the generation of a database of all the theoretical peptide masses grouped by proteins (hereinafter, reference protein database).

### 3. matching of the acquired spectrum against the generated reference protein database

From the acquired spectrum a list of peptide masses (hereinafter, query mass list) is extracted [5-7] and compared with those of each protein contained in the reference protein database. Due to the intrinsic measurement error, the comparison between reference masses and experimental data has to be made considering a tolerance window around the experimental mass value, expressed in term of absolute or relative mass. The evaluation for each protein of the agreement between experimental and theoretical spectra can be done by several algorithms available in the literature [8-12] and in some cases already implemented in software tools (e.g., Aldente [13], Mascot [14], Ms-Fit [15], Piums [16] and ProFound [17]). The output of these algorithms is a ranked list (hereinafter, ranked candidate protein list) of all the proteins of the reference database, which have at least one peptide whose mass matches against those of the query mass list. Finally, a statistical validation of the results should be performed, leading to the retrieval of a very short list of proteins (hereinafter, significant candidate protein list) that match with high confidence the protein(s) actually contained in the sample [18].

Even if PMF is conceptually simple and fast, it presents some liability, mainly due to the occurrence of false positives in the final list, especially when the sample is a mixture of proteins and the reference database is wide. The available tools do not provide complete satisfactory strategies. All tools present some limitations and no one implements all the best solutions proposed in the literature. They have a closed architecture and than they are not modifiable by adding other more effectiveness solutions. The main problems concern the possibility of choosing the reference protein database and to update it, of choosing an adeguate maximum number of missed cleavages or the post-translational modifications of interest (see the Methods section for their explanations). Usually, masses of contaminants detected by the spectrometer are not removed before identifications or contaminant proteins cannot be specified by the user. Ranking algorithms can be improved and statistical evaluation of the results are frequently lacking. This paper presents a complete Perl procedure for the analysis of mass spectrometry data by PMF, starting from the creation of the reference protein database and ultimately providing the statistical validation of the results. The three scoring methods reported in [12] were chosen and implemented in the MsPI Perl tool. Their performance has been tested on an experimental dataset acquired on ten protein bands. The refined output provided by MsPI has been benchmarked to the results obtained on the same dataset using two on line available tools, Piums and Mascot, and this evaluation will be presented in this paper. While Mascot is one of the most commonly used software tools for protein identification, its scoring method, however, could not be implemented into MsPI because of the lack of documentation.

## Methods

This section describes the procedure proposed in this paper to analyze PMF data and implemented into the MsPI Perl scripts. It includes the downloading from the web of a protein database and its *in silico *digestion to build a reference protein database, the removal of contaminants from the query mass list, the definition of a ranked candidate protein list and the extraction of a shorter significant candidate protein list.

### Reference protein database

A reference database, containing the masses of all possible peptides obtained by the enzymatic digestion of all known proteins with a selected protease, is created. First of all a suitable protein database is downloaded and then elaborated as described in the following paragraphs for obtaining the theoretical spectra of every protein stored in the database. For example, in this work the Swiss-Prot database, available on the European Bioinformatics Institute web site [19], was considered. Release 56.1 of September 2008 contains 397.539 complete protein sequences belonging to different organisms (20.326 belong to Homo sapiens). A Perl script was created for automatically downloading the last available release (see the Appendix).

Amino-acid sequences of Swiss-Prot protein entries are provided in a FASTA format file. To make the matching phase more efficient, the number of proteins submitted to *in silico *digestion is reduced by restricting the search to those protein entries belonging just to one or few organisms of interest. In addition, the information coded in the header line of the FASTA format is decoded and efficiently organized through a Perl regular expression. Moreover, MsPI completes the downloaded database with several additional information, such as protein MWs and pIs. These data can help further reducing the candidate proteins on the basis of the "qualitative" information about the MW and the pI coming from the electrophoretic gel, thus speeding up the searching step and/or decreasing the number of false positives.

#### Computation of molecular weight and isoelectric point

In the considered version of the Swiss-Prot database, the MW and the pI of proteins are not part of the annotations, so that they have to be computed by MsPI. The MW of a protein can be computed on the basis of its amino-acid sequence, knowing the average mass of each amino acid. In fact, it is sufficient to add, in accordance with the protein sequence, the average amino-acid weights reported in Table 1 to the average mass of one water molecule (*H*_2_*O*_*MWav *_= 18.01524) to take into account that the amino- and carboxyl- terminals are not involved in any peptide bond.

**Table 1:** The amino acids and their monoisotopic and average masses used for computing the molecular weight of a protein or a peptide are reported (source [20]).

Amino acid		Monoisotopic mass	Average mass
Alanine	A	71.04	71.08
Arginine	R	156.10	156.19
Asparagine	N	114.04	114.10
Aspartic acid	D	115.03	115.09
Cysteine	C	103.01	103.14
Glutamic acid	E	129.04	129.12
Glutamine	Q	128.06	128.13
Glycine	G	57.02	57.05
Histidine	H	137.06	137.14
Isoleucine	I	113.08	113.16
Leucine	L	113.08	113.16
Lysine	K	128.09	128.17
Methionine	M	131.04	131.19
Phenylalanine	F	147.07	147.18
Proline	P	97.05	97.12
Serine	S	87.03	87.08
Threonine	T	101.05	101.11
Tryptophan	W	186.08	186.21
Tyrosine	Y	163.06	163.18
Valine	V	99.07	99.13

Therefore:



where *amw*_*i *_is the average mass of the *i-th *amino acid and *N *is the total number of amino-acid residues of the protein.

The pI of a protein is the value of pH at which the molecule does not carry any net charge [20]. It depends on the molecular composition of the protein and in particular on the presence of amino-acid residues carrying a charge. Therefore, the pI does not depend on the whole amino-acid sequence, but only on the seven amino acids that have ionizable groups in the side chain and on the amino- and carboxyl- terminals. For computing pI, the pKr value of each amino acid must be known. The pKr is formally given by:



where *Kr *is the acid dissociation constant of the residue, that is the degree of dissociation of the molecule in water solution. The determination of the pKr coefficients is not a trivial task. In fact, as reported in Table 2, there is not a wide consensus in the literature and in the different software tools about the pKr values [21,22]. Fortunately, the computation of the pI value of a protein is not a crucial step of the identification procedure, because it does not affect the ranking in the candidate list. In fact, pI is used to filter out some of the false positive identifications on the basis of its experimental value read from 2D gel. Due to the uncertainty in its computation and the experimental noise the comparison is usually made with a wide tolerance. In the MsPI package, in accordance with other tools, the values suggest by Lehninger were adopted, but the user can set the values proposed by the other authors as well.

**Table 2:** The amino acids and the terminal groups that influence the pI of a protein with their charge polarity are shown. Four pKr values are tabulated for each of them, as defined by Lehninger [21], Solomon [22], Sillero [22], Rodwell [22]. In this work, Lehninger's values were used.

Amino acid	Polarity	pKr Lehninger	pKr Solomon	pKr Sillero	pKr Rodwell
Glutamic acid (E)	-	4.25	4.30	4.50	4.25
Aspartic acid (D)	-	3.65	3.90	4.00	3.68
Cysteine (C)	-	8.18	8.30	9.00	8.33
Tyrosine (Y)	-	10.07	10.10	10.00	10.07
Histidine (H)	+	6.00	6.00	6.40	6.00
Lysine (K)	+	10.53	10.50	10.40	11.50
Arginine (R)	+	12.48	12.50	12.00	11.50
Carboxyl terminal (COOH)	-	3.10	2.40	3.20	3.10
Amino terminal (*NH*_2_)	+	8.00	9.60	8.20	8.00

For the amino acids with positive polarity, the fraction of charged molecules can be computed as:



Whereas, in case of negative polarity the fraction of charged molecules can be computed as:



The total net charge of a protein is then given by the sum of all the charges carried by all the amino-acid residues. Therefore:



where the Greek letters represent the number of occurrences of each kind of amino acid. The pI can be then computed letting *Q*^*prot *^= 0 and resolving the equation with respect to the variable pH. This equation does not admit an analytical solution, then it has to be solved through an iterative algorithm (e.g. a bisection method).

#### *In silico *enzymatic digestion

After MW and pI computation, the *in silico *enzymatic digestion is performed. In the "real world", the enzymatic digestion is a chemical reaction that causes the fragmentation of the protein at specific positions of the peptide chain. There are several proteases that catalyze this reaction. In MsPI the model of trypsin (bovine or porcine), one of the enzymes most frequently used in PMF, was implemented, but other proteases can be easily added. As a rule, trypsin cleaves the protein when it meets the C-side of the amino acid lysine (K) or arginine (R) involved in a peptide bond. Nevertheless, there are some cleavage exceptions due to the presence of particular amino acids around the cleavage site (see Table 3) [23]. A Perl regular expression was implemented to simulate the cleavage rules.

**Table 3:** The exceptions to the basic cleavage rules of the trypsin are shown: *AA*_-1 _is the amino acid immediately preceding the K or R amino acid, *AA*_0 _is the last amino acid before the cleavage site (K or R) and *AA*_1 _is the first amino acid after the cleavage site. In these cases, trypsin does not easily cut the amino-acid chain (Source [23]).

*AA*_-1_	*AA*_0_	*AA*_1_
not W	K	P
not M	R	P
C	K	D
D	K	D
C	K	H
C	K	Y
C	R	K
R	R	H
R	R	R

For each peptide obtained by *in silico *digestion, the theoretical monoisotopic MW is then computed as:



where 18.01056 is the monoisotopic weight of one water molecule, *mmw*_*i *_is the monoisotopic mass of the *i-th *amino acid and *N *is the total number of amino-acid residues of the peptide. Additional information required by PMF identification procedure is associated to the peptide: a peptide identification code (ID) and its position in the "parent" protein (i.e. start and stop amino acids), which will be used for computing the total amino-acid coverage.

#### Missed cleavages and post-translational modifications

Being the protein digestion a stochastic process, the protease does not cleave the peptide bond at every occurrence of the cleavage rule and therefore some cleavages along the protein can be randomly missed (missed cleavage – MC). So, after the digestion process there are some longer peptides, those which have some MCs. They include, in a single peptide, two or more consecutive short peptides of the protein. Therefore, the digestion of a protein that contains *n *- 1 cleavage sites, can theoretically produce *n*(*n *+ 1)/2 different peptides. Even if the database should contain all the possible generated peptides, to avoid an uncontrolled growth of the database size and consequently of the computational complexity of the search, it is reasonable to fix the maximum number of admitted consecutive MCs, considering that a great number of consecutive MCs is hardly probable. Note that fixing this number to *m*, the database grows of about a factor *m *instead of a factor (n+ 1)/2. Being in general *m *<<*n *the advantage is evident.

Furthermore, the reference protein database has to take into account also the post-translational modifications (PTMs), i.e. chemical modifications of specific amino acids affecting the MW, which occur in cells (e.g. phosphorylations) or are introduced during the sample preparation procedure (e.g. oxidations). Usually, PTMs can be considered either fixed or variable: in the former case, the PTM is present at every occurrence of the respective amino acid, whereas in the latter one the modification occurs randomly and then may or may not be present. A PTM causes the variation of the peptide MW, increasing or decreasing it. For example, when the carboxyamidomethylation of the amino acid cysteine occurs, the MW of this amino acid increases of about 57.02 Da. Again for computational reasons, the maximum number of variable PTMs that can occur on the same peptide has to be fixed *a priori*.

To create a database that reflects the "real world" as much as possible, all the combinations of MCs and PTMs have to be generated and included in the reference protein database.

#### Database files

After *in silico *digestion of all the stored proteins, the resulting peptides are in turn stored into a new suitable reference protein database. To speed up the PMF searching step, this new database is split into multiple files grouping together the peptides with similar MWs, following heuristic rules based on the distribution of the peptide MWs. For example, for the *MW *≤ 500, five files are created, one for peptides whose MWs range are from 0 to 100 Da, one for peptides from 100 to 200 and so on.

This strategy allows to read during the searching step only those files that could contain peptides matching one of the masses of the query mass list.

### From the query mass list to the ranked candidate protein list

To identify the proteins in the biological sample, the masses obtained after the pre-processing and the peaks extraction of the acquired spectrum [6] have to be matched against those in the reference protein database. However, the direct use of the mass list is not appropriate, because some contaminant masses introduced during sample preparation could be present. As reported in [24] the main contaminant sources are:

• the protease used in the enzymatic digestion (for example trypsin);

• human skin and dandruff keratins:

1. K1 – Keratin, type II cytoskeletal 1 (Cytokeratin-1);

2. K2E – Keratin, type II cytoskeletal 2 epidermal (Cytokeratin-2e);

3. K9 – Keratin, type I cytoskeletal 9 (Cytokeratin-9);

4. K10 – Keratin, type I cytoskeletal 10 (Cytokeratin-10);

• the MALDI matrix and the electrophoresis dye.

The enzymatic contaminants come from the autolysis process of the protease itself used to digest proteins. They depend on the total amount of protease used and on the reaction time. The keratins, instead, come from the scientist(s) performing the different sample preparation steps and often they are the most important contaminants. The exclusion of these contaminant masses from the query mass list is often fundamental for a successfully identification.

#### Contaminant mass removal

The removal of contaminant masses is not a trivial task and a well-established procedure is not yet available in the literature. The main difficulty is that the removal step may eliminate also an important part of the "true signal", sometimes leading to a worsening of the overall identification capabilities. In fact, a contaminant mass could be isobaric to that of a peptide of the sample. Therefore, starting from these considerations, in this paper the following procedure is proposed and was implemented in the software tool. First of all, the list of the possible contaminant masses was generated by the *in silico *digestion of the considered contaminants (see previous paragraph). To limit the risk of removing some masses belonging to the proteins under investigation, only those masses in the removal mass list that are not very frequent in the reference protein database (i.e., those that are hardly probable to come from proteins other than contaminants) were really removed from the query mass list. The problem became the quantification of this probability threshold. Even if this threshold can be considered as a design parameter that can be tuned by the user, a simulated analysis was performed in order to define a reasonable value. A set of 100 simulated human proteins was generated: each protein item was randomly extracted from the database and the corresponding peptides were considered. For every protein, a set of contaminant peptides was included and, finally, a Gaussian noise was added to each peptide mass. The 100 new proteins were then identified by applying a mass tolerance equal to the standard deviation of the above Gaussian noise and the frequency threshold was varied from 10^-4 ^to 10^-7^. On the basis of the simulated analysis just described and of the experience, a reasonable value for the threshold relevant to human protein identification seems to be 10^-5^. Conversely, because contaminant masses due to the ionization of the MALDI matrix and electrophoresis dye are present in the spectrum at lower values (< 900 Da), where only short peptides are present, usually, the lower mass values are discarded. Similarly, because long peptides (over 5000 Da) generated by trypsin are extremely rare and then the acquired signal is mainly due to the noise, an upper bound value (around 4000 to 5000 Da) is also fixed in the spectrum acquisition mass range.

#### Scoring methods

After the contaminant mass removal, the query mass list is ready to be compared with the masses of the reference database (searching step). A match between the query mass *x*_*l *_and the reference mass *y*_*j *_occurred if:



where *δ*_*l *_is the mass tolerance for mass *x*_*l*_. For each protein in the reference database the number of matches is calculated and a scoring function is used to evaluate the global similarity between the candidate protein and the experimental data. In a probabilistic context, this function represents the probability that the considered protein has generated the experimental data (i.e. the likelihood). In the literature there are many papers that propose several scoring methods for the PMF approach. In this work the methods proposed by Samuelsson and colleagues have been considered and implemented [12]. They define three different scores, based on different hypotheses on the mass tolerance and on the distribution of the peptide masses in the reference database. The mass tolerance is assumed as either absolute (in dalton) or relative (in ppm), whereas the mass distribution can be either uniform or not uniform. Table 4 summarized the hypotheses made by the three scoring methods.

**Table 4:** The hypotheses under the three scoring methods proposed by Samuelsson et al. are shown.

Method	Mass tolerance	Peptide mass distribution
1	Absolute (Da)	Uniform
2	Relative (ppm)	Uniform
3	Relative (ppm)	Not uniform

Note that an implementation of Samuelsson's scores already exists within the Piums software tool [16]. However, as declared in [12], only the first scoring method has been really implemented and tested. Therefore, it is wrongly used by Piums when the relative mass tolerance is chosen by the user. To fill this gap and to better explore all the possible situations, all the three scores were implemented in the MsPI software tool.

#### Method 1

The probability to have at least *r *matches out of *L *query masses can be computed by the Binomial distribution, so that:

(1)

*p *is the probability that for a given protein a mass matches at least once in the reference database. Since method 1 assumes an absolute tolerance (*δ*) and a uniform distribution of the masses in the whole acquisition mass range (Δ), *p *does not depend on the specific query mass as required by the Binomial assumption and it is equal to 2*δ */Δ.

#### Method 2

The likelihood for an observed set of matches against a random protein can be computed as:

(2)

where *p*_*l *_is defined similarly to the *p *of the method 1 and *ϕ*_*l *_is equal to 1 if the mass *x*_*l *_has at least one match in the reference database, 0 otherwise. Note that, in this case, *p*_*l *_depends on the specific query mass, so that the Binomial distribution cannot be used. In fact, since method 2 assumes a relative tolerance (*δ*_*l*_) and a uniform distribution of the masses, *p*_*l *_is equal to 2*δ*_*l*_/Δ or equivalently 2*ppm x*_*l*_/Δ.

#### Method 3

It does not assume that the distribution of the peptide masses in the reference database has to be uniform, instead in general it can be a function of the peptide masses and of the number *N *of peptides in the same protein. The probability that *r *of the *N *peptides of a protein match the query mass list can be again computed by the Binomial distribution:

(3)

where *λ *is the probability that a peptide of the candidate protein matches at least one of the *L *query masses and it is given by:



with *p*_*l *_equal to the probability of randomly matching the mass *x*_*l*_. It can be computed by integrating the mass density probability function *ρ*(*MW*|*N*) as:

(4)

The global similarity score on which candidate proteins are ranked is finally computed as minus the natural logarithm of the (1) or (2) or (3), so that a protein with a low probability for random matches has a high score. The higher the score, the greater is the probability that the considered protein is really present in the sample.

Note that after defining *δ*_*l *_and Δ, scores 1 and 2 are immediately computable, while score 3 requires to define *ρ*(*MW*|*N*) and to compute the integral (4). In this paper, two strategies are proposed to this aim: the first one assumes *ρ*(*MW*|*N*) to be uniform on all the considered range of masses (Δ) and independent from *N*, the second one tries to model the dependence of *ρ*(*MW*|*N*) from the two variables. In the first case it is trivial to verify that *p*_*l *_= 2*δ*_*l*_/Δ, whereas in the second case the *ρ*(*MW*|*N*) has to be learned from the reference database, discretizing both the mass variable and *N*. Therefore, a discretization grid (with *N *on the rows and *MW *on the columns) was built up and several classes were defined. To obtain a reasonable distribution among the classes they were defined considering the percentiles of each of the two variables. Then the probability of the (*i*, *j*) class was computed as:



and then the probability density as:



where Δ*m*_*j *_and Δ*N*_*i *_were the ranges in the (*i*, *j*) class of the masses and of N, respectively. Given *x*_*l*_, *δ*_*l *_and *N*, the probability *p*_*l *_is given by:



where *b*_*j *_is the mass value that separates the classes *j *and *j+1*.

#### Statistical significance of the score

The protein list generated in the previous step ranks all the proteins in the reference database that match at least one query mass on the basis of one of the similarity score chosen by the user. However, the score itself does not provide a threshold in order to retrieve a reasonable subset of candidates. Sometimes, this is done by fixing an arbitrary threshold in order to obtain a "short" list of the desired length. In a statistical framework, this problem becomes to define a threshold score that is able to reject with a desired degree of certainty the null hypothesis that the score is not greater than the one obtained by chance [25].

To this aim a database of random sequences can be built up. Then, the identification process already performed in the reference database, has to be repeated on this random database for assigning a significance value to each score in the ranked candidate protein list, as proposed in [12]. Starting from probability *P*_*rnd*_, i.e. the probability that a score greater than or equal to a fixed value is obtained when the query mass list is compared with a protein of the random database, two indexes are defined: the p-value and the quality index. The p-value is the probability to achieve in a random database of the same size of the reference database a score greater than or equal to that considered. The p-value is given by 1 - (1 - *P*_*rnd*_)^*D*^, where *D *is the number of proteins of the reference database. The quality index, instead, indicates how much the random database should be larger than the reference database to observe the same number of proteins with a score greater than or equal to a fixed value. The score, the p-value and the quality index can be computed for each of the candidate proteins.

Given the reference database of *D *proteins, the random database of *D*_*rnd *_sequences is generated in the following way. First, *D*_*rnd *_proteins are sampled with replacement from the reference database, then they are digested *in silico *and the peptide masses derived from the protein items having the same number of peptides are pooled together. Finally, for each extracted protein a random protein is built by randomly sampling with replacement the associated pool of peptide masses. For obtaining an accurate evaluation of the p-value, the random database size *D*_*rnd *_must be chosen sufficiently large. *D*_*rnd *_is another use parameter.

## Results and discussion

The MsPI software tool, described in details in the Appendix, was tested using a Sun W1100z workstation with Linux SUSE distribution and the results have been compared with those obtained using two other software tools available on line.

### Dataset

The dataset used for testing MsPI contains 10 gel bands of human proteins, already analyzed in a previous work by Troiani and colleagues [26] using both PMF and PFF approaches. The proteins in the biological sample were separated by a 1-D gel following a standard protocol and the enzymatic digestion was performed with bovine trypsin. The peptides obtained were then analyzed with a MALDI-TOF mass spectrometer using the workstation Voyager DE_PRO (Applied Biosystems) and the acquisition mass range was set to 900–4000 Da. The peak list of each band of this dataset was stored in an ASCII file.

### Reference protein database

The reference protein database was generated starting from Swiss-Prot release 56.1 (September 2008).

In accordance to [26], protein identification was carried out imposing these conditions:

• organism: Homo sapiens;

• up to two consecutive MCs allowed;

• fixed carboxyamidomethylation of the amino acid cysteine;

• variable oxidation of the amino acid methionine (maximum two for peptide).

The fixed PTM at cysteine causes an increase of the amino acid mass of about 57.02 Da. The oxidation at methionine causes a mass increase of about 15.99 Da.

As discussed in the Methods section, the mass range considered in the analysis was 800–5000 Da, which is quite similar to the mass spectrometry acquisition range set by Troiani and colleagues.

The random database generated for the validation contains 100.000 proteins (about 5 times greater than the reference one). These two databases take up 252 MB and 1 GB of disk space, respectively.

### Contaminant mass removing

The contaminant masses were removed from the query mass list in accordance to the implemented procedure. In addition to keratins K1, K2E, K9 and K10, the bovine trypsin was included in the contaminant list. The frequency threshold was set to 10^-5^. The number of query masses of the 10 bands before and after the contaminant mass removing are shown in Table 5. Results depend on the mass tolerance used, even if a fixed tolerance of 0.3 Da or a relative tolerance of 100 ppm provide very similar results. It is interesting to note that, when the protein identification procedure was applied to the removed masses, keratins K1, K2E, K9 and K10 were identified as significant. That proves the real presence of these contaminants in the analyzed samples as discussed in [24].

**Table 5:** The number of query masses in each sample band before and after contaminant mass removing by MsPI routine are shown.

Band	Number of query masses	Mass tolerance	
		0.3 Da	100 ppm
1	121	64	65
2	111	72	72
3	123	73	74
4	106	55	55
5	164	98	99
6	172	116	116
7	123	62	62
8	71	39	38
9	175	119	120
10	52	27	28

### Protein identification

All the scoring methods implemented were tested and results were compared with those achieved by Piums and Mascot. Both absolute and relative mass tolerance were used. In particular, they were fixed to 0.3 Da and 100 ppm, respectively. The p-value cut off imposed for the statistical validation of the results was 0.05.

The query mass list obtained after the contaminant mass removing was used as input for both Piums and Mascot, because they do not implement contaminant removal capabilities. A summary of the results of the overall analysis is shown in Table 6, where for each band is indicated the position in the ranked list of the "true" protein, the number of statistical significant proteins when the MW of the gel band was either considered or not, the MW and the pI of the "true" protein estimated by the software tools, the number of matched masses, and the sequence coverage defined as the ratio of the number of amino acids of the matched peptides to that the whole protein. For each band, the sequence coverage was between 25% and 50%, independently from the considered score and tool, except for band nine that had a higher coverage (about 75%).

**Table 6:** For MsPI 1, Mascot 1 and Piums the mass tolerance was set to 0.3 Da; in the other cases it was set to 100 ppm. For MsPI 1, MsPI 2 and MsPI 3, score 1, 2 and 3 were respectively used with uniform distribution. For each band the position of the "true" protein in the significant candidate list, the length of that list either using or not using the knowledge about the MW of the band, the MW and the pI of the "true" protein, the number of matching masses and the sequence coverage are reported.

Gel band (#)	Software tool	Rank (# significant proteins without MW filtering)	Rank (# significant proteins with MW filtering)	MW (Da)	pI	Matches (#)	Coverage (%)
1	MsPI 1	2 (2)	1 (1)	123852	5.31	23	0.267
	MsPI 2	7 (8)	1 (1)	123852	5.31	23	0.270
	MsPI 3	2 (2)	1 (1)	123852	5.31	23	0.267
	Mascot 1	1 (1)	1 (1)	124292	5.50	23	0.250
	Mascot 2	1 (1)	1 (1)	124292	5.50	23	0.250
	Piums	-	- (-)	-	-	21	0.242

2	MsPI 1	1 (2)	1 (1)	191785	5.32	36	0.252
	MsPI 2	1 (4)	1 (1)	191785	5.32	35	0.252
	MsPI 3	1 (2)	1 (1)	191785	5.32	35	0.252
	Mascot 1	1 (1)	1 (1)	193260	5.48	36	0.250
	Mascot 2	1 (3)	1 (1)	193260	5.48	35	0.250
	Piums	1 (1)	-	-	-	36	0.257

3	MsPI 1	2 (8)	1 (1)	104981	5.08	21	0.245
	MsPI 2	- (4)	- (-)	104981	5.08	21	0.245
	MsPI 3	2 (4)	1 (1)	104981	5.08	21	0.245
	Mascot 1	1 (2)	1 (1)	105245	5.27	21	0.240
	Mascot 2	1 (6)	1 (2)	105245	5.27	21	0.240
	Piums	- (-)	-	-	-	19	0.254

4	MsPI 1	2 (4)	1 (1)	95382	6.38	20	0.286
	MsPI 2	1 (5)	1 (1)	95382	6.38	20	0.286
	MsPI 3	1 (6)	1 (2)	95382	6.38	20	0.286
	Mascot 1	1 (1)	1 (1)	96246	6.41	22	0.320
	Mascot 2	1 (1)	1 (1)	96246	6.41	22	0.320
	Piums	- (-)	-	-	-	19	0.341

5	MsPI 1	2 (8)	2 (3)	84351	4.70	28	0.418
	MsPI 2	3 (3)	2 (2)	84351	4.70	28	0.418
	MsPI 3	2 (12)	2 (3)	84351	4.70	28	0.418
	Mascot 1	1 (2)	1 (2)	83554	4.97	35	0.480
	Mascot 2	1 (3)	1 (2)	83554	4.97	34	0.480
	Piums	1 (1)	-	-	-	28	0.364

6	MsPI 1	2 (8)	1 (1)	67900	7.37	24	0.515
	MsPI 2	- (1)	- (-)	67900	7.37	23	0.509
	MsPI 3	1 (6)	1 (1)	67900	7.37	23	0.509
	Mascot 1	1 (2)	1 (1)	68519	7.58	24	0.510
	Mascot 2	1 (4)	1 (2)	68519	7.58	23	0.500
	Piums	- (-)	-	-	-	19	0.446

7	MsPI 1	1 (3)	1 (1)	53146	4.82	21	0.468
	MsPI 2	1 (2)	1 (1)	53146	4.82	21	0.468
	MsPI 3	1 (2)	1 (1)	53146	4.82	21	0.468
	Mascot 1	1 (1)	1 (1)	53676	5.06	21	0.500
	Mascot 2	1 (3)	1 (1)	53676	5.06	21	0.500
	Piums	- (-)	-	-	-	16	0.413

8	MsPI 1	- (2)	- (-)	49674	4.53	8	0.232
	MsPI 2	- (1)	- (-)	49674	4.53	8	0.232
	MsPI 3	- (1)	- (-)	49674	4.53	8	0.232
	Mascot 1	- (-)	- (-)	50095	4.78	8	0.230
	Mascot 2	- (-)	- (-)	50095	4.78	8	0.230
	Piums	- (-)	-	-	-	8	0.232

9	MsPI 1	1 (4)	1 (1)	45982	9.79	31	0.784
	MsPI 2	1 (4)	1 (1)	45982	9.79	32	0.787
	MsPI 3	1 (5)	1 (1)	45982	9.79	32	0.787
	Mascot 1	1 (1)	1 (1)	46180	9.45	31	0.730
	Mascot 2	1 (1)	1 (1)	46180	9.45	31	0.730
	Piums	1 (1)	-	-	-	27	0.715

10	MsPI 1	1 (9)	1 (2)	41611	5.10	13	0.349
	MsPI 2	2 (5)	2 (2)	41611	5.10	13	0.379
	MsPI 3	1 (6)	1 (4)	41611	5.10	13	0.379
	Mascot 1	1 (4)	1 (4)	42052	5.29	13	0.370
	Mascot 2	1 (4)	1 (4)	42052	5.29	13	0.370
	Piums	2 (2)	-	-	-	13	0.380

Note that, since the electrophoretic gel of this study is 1-D, no information about the pI was available.

#### Comparison between MsPI and Piums

In Piums only the scoring method 1 was implemented. It supposes an absolute mass tolerance and a uniform distribution of the peptide masses, even if the implemented user interface wrongly allows the user to set a relative tolerance. In addition, Piums does not allow to consider two MCs: only one MC can be set. Moreover, the Swiss-Prot protein database available within the tool is an old version (44.6) and it cannot be updated.

Using an absolute mass tolerance of 0.3 Da, MsPI with the scoring method 1 includes in the list of the significant proteins the "true" protein nine times over ten, while Piums reaches the same result only for four bands. The lower performance of Piums is probably due to the fact that only one MC is allowed. Moreover, as Table 6 shows, Piums does not provide the user with the MW and the pI of the candidate proteins. This could be a problem for the analyst that cannot use the electrophoretic information to better identify the band.

#### Comparison between MsPI and Mascot

Looking at the results obtained when the mass tolerance was fixed to 0.3 Da, MsPI with the scoring method 1 and Mascot (Mascot 1 in Table 6) include nine times over ten the "true" proteins in the significant candidate protein lists. The number of significant proteins included in the list is in general higher for MsPI than for Mascot (overall 50 proteins against the 15 of Mascot), but if the significant results are filtered in accordance to the MW read from the gel, the lists shorten mainly for MsPI, reaching an overall length of 12 and 13 for MsPI and Mascot, respectively.

The results obtained using MsPI (MsPI 3 in the Table 6) and Mascot (Mascot 2 in Table 6) with a relative mass tolerance (100 ppm) show that both methods include nine times over ten the "true" proteins in the candidate lists and that Mascot finds less significant proteins than MsPI. However, also in this case, when the MW is used to reduce the size of the candidate lists, MsPI obtains less significant proteins (14 against 15) minimizing the number of false positives.

As a final remark, note that, at least in this study, the significant candidate lists obtained by MsPI and Mascot share only the "true" proteins showing that the two identification algorithms are quite different.

#### Comparison between the MsPI scoring methods

Comparing the scoring methods implemented in MsPI on our dataset, the following considerations can be made. First of all, scoring method 3 with a not uniform distribution of peptide masses seems to be not very effective, because it attributes higher scores to very long proteins. In fact, no band was correctly identified, i.e. the "true" protein was never included in the significant candidate lists (results not reported). Scoring method 2 shows a reasonable performance, in fact it includes seven times over ten the "true" protein in the significant candidate lists. In this study, score 1 and 3 show the best performances, correctly "identifying" nine band over ten.

Note that band 8 was not identified by any of the software tools tested (i.e., MsPI, Piums and Mascot) and actually a more informative analysis using the PFF approach was required to identify it [26].

For seven bands MsPI indicates only one significant candidate when the MW information was considered, allowing a complete determination of the unknown protein. Conversely, in two cases (band 5 and 10) the lists include more than one candidate. Interestingly, the significant candidate proteins have similar characteristics both for the MW and pI and they belong to the same family. In fact candidate proteins, when aligned by the Needleman-Wunsch algorithm implemented in the Bioinformatics Toolbox of Matlab (version 2.5), showed a high similarity: for band 5 the identity between the two candidates was 86% and the similarity was 96%, while for band 10 they were about 99% and 100%, respectively.

### Computational performance of MsPI

The routines *swiss2MsPI.pl*, *create_database.pl *and *create_database_random.pl*, used to create the reference protein database and the random database, took 30, 1014 and 7747 seconds, respectively (see the Appendix for a detailed description of the MsPI routine structure). They have to be executed once or when the main parameters of the analysis are modified (e.g, the organisms of interest, the considered PTMs and MCs, and so on). In fact, the generated databases are stored in the *db *subfolders.

For what concerns the third step of PMF, the time required for the identification of a band depends on the number of query masses and on the scoring method adopted. In the study considered in this paper, after a careful optimization in the implementation of the searching procedure band identifications took about one minute on average, whereof 2/3 of the time was used for the evaluation of the statistical significance of the results. Scoring method 2, which uses a variable mass tolerance and a uniform database distribution, employed more time than the other two methods (it does not use the Binomial distribution). The estimation of the peptide mass distribution made by the routine *make_grid.pl *and used for the implementation of score 3 required 130 seconds for the reference database and 2420 seconds for the random database. For this reason it is stored after its first computation.

## Conclusion

In this paper, a complete procedure for protein identification by PMF has been proposed. This procedure starts from a query mass list leading to the generation of a candidate protein list and includes the removal of contaminant masses and the statistical validation of the results. The procedure has been fully implemented in a Perl software tool, called MsPI, available for free downloading and briefly described in this paper. The principal goal of MsPI is to implement an exhaustive procedure that:

1. creates a reference database in which the unknown protein is searched;

2. performs that search;

3. computes a similarity score for each protein hit retrieved;

4. creates a random database for a statistical validation of the results.

Many software tools are available in the literature for solving the problem of protein identification, however, all of them either do not implement all the above described steps or present some limitations in their applicability. The software tool proposed here attempts to provide a complete and customizable solution to this task.

The reference protein database is created with particular attention to the *in silico *reproduction of all processes that occur during the enzymatic digestion and the sample preparation. For example, maximum flexibility is left to the user in the definition of PTMs and of the maximum number of MCs.

Several scores, based on different hypotheses, were made available in the tool for ranking the proteins that have one or more matches with the query mass list. In particular, both an absolute and a relative mass tolerance can be specified for the evaluation of the matches/mismatches between two masses. Since the problem was formulated in a probabilistic context, it is also possible to associate a level of statistical significance to each protein in the candidate list.

A software tool called Piums was already present in the literature and it implements a similar probabilistic score. However, the comparison between MsPI and Piums has highlighted that MsPI is more flexible, in that the reference protein databases can be updated and more than one consecutive MC can be considered. Moreover, Piums does not allow to set relative mass tolerance using a suitable score and it is not customizable by the user (e.g. no arbitrary PTMs can be considered). Further, at difference from Piums, MsPI provides the user with the MW and the pI values of all the candidate proteins, helping the analyst to identify the correct proteins in a gel band or spot.

Some comparisons were made also with respect to Mascot, one of the most popular softwares in this field. The overall performance of MsPI and Mascot were very similar, even if some differences were present in the candidate lists. MsPI retrieves a higher number of significant results, and consequently more false positives, than Mascot, nonetheless by exploiting the knowledge about the MW of the band MsPI provides a lower number of candidate proteins. As MsPI, Mascot also computes the MW and the pI of the candidate proteins, even if some slight differences were found.

Since the two softwares implement different algorithms and their candidate lists shared in general only the "true" protein, running both methods on the same query list and then comparing the output could be an interesting opportunity. The intersection of the two candidate lists should improve the quality of the results.

While Mascot and Piums do not implement the relevant utility, the removal of the contaminant masses from the acquired spectra is however an important step of the PMF analysis. In fact, by performing the protein identification analysis on the masses removed from the query mass list by the right MsPI algorithm we could highlight the real presence of keratin contaminants in the biological samples. Moreover, the analysis performed with Mascot on the whole query mass list without removing contaminants showed that many bands were identified as mixture of keratins and other proteins, confirming again the sample contamination.

Comparing the different scoring methods implemented in MsPI, it turns out that scoring methods 1 and 3 with a uniform distribution have the best performances, identifying nine bands over ten. The scoring method 2 identifies seven bands over ten, whereas the method 3 with a not uniform distribution shows some liability.

MsPI provides for each candidate protein the significance of the score allowing a natural cut off on the candidate list. Mascot, instead, defines a threshold on the score on the basis of the number of sequences of the reference database: if the score is above this threshold, the considered protein is significant. Moreover, it optionally provides the number of proteins over this threshold in a randomly generated database.

When the statistical validation process is performed, four cases could show up:

1. the protein with the best score is the only significant one;

2. more than one significant proteins are in the list and one of them is the "true" protein;

3. the "true" protein is not significant, but there are other significant proteins;

4. there is no significant protein.

When case 2 occurs, the electrophoresis experiment can be crucial for protein identification in MsPI, since often the proteins identified as significant and yet wrong have a high MW. So, the electrophoresis information allows to reasonably drop some of the candidates. Conversely, in Mascot it was observed that the hits marked as significant often have the same MW of the "true" one, therefore the gel information becomes less useful.

When either the "true" protein is not included in the list of significant results or no statistically relevant results are obtained, the failure in the identification process may be due to different reasons, such as issues with sample preparation or the presence of a substantial number of contaminant peaks in the spectrum. In order to get an answer and preferably the right one in these cases, the analyst can resort to a PFF experiment, which relies on reconstructing the sequence of a defined number of product peptides to identify the parent protein.

## Availability and requirements

**Project name**: MsPI

**Project home page**: 

**Operating system(s)**: Platform independent

**Programming language**: Perl

**Other requirements**: The following modules are required: Cwd, Lwp, IO::ZLib, Math::BigInt, Math::BigFloat (available via CPAN)

**Any restrictions to use by non-academics**: A written authorization has to be required to the correspondent author.

## Appendix

### MsPI implementation

**Figure 2:**
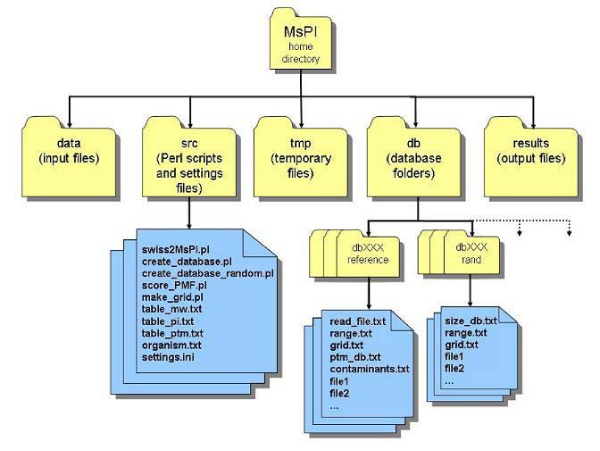
**The directory structure created during the installation of the MsPI tool**.

MsPI (Mass spectrometry Protein Identification) is the Perl software tool that implements the methodology described in this paper. Perl was chosen to implement the second and the third steps of PMF, for its capabilities in alphabetic strings manipulation, which are the main structure for representing proteins and peptides, thanks to the power of its regular expressions. MsPI is a collection of several Perl scripts and ASCII files distributed in several directories. The directory tree created by MsPI is shown in Figure 2. The *data *folder contains the query mass list and a configuration file specifying some user-dependent parameters required by MsPI. Details are reported on the MsPI web site in the README file included in the software distribution.

The *tmp *folder contains some compressed temporary files created from Perl routines, such as the protein database downloaded from the network.

The *db *folder contains the subfolders within the reference and random databases created in accordance to the user's choices. Several databases with different characteristics can be present at the same time in different subdirectories.

The *results *folder contains the output of the identification procedure, that is the candidate protein list. The *src *folder contains the Perl routines and some additional ASCII files that can be complemented or modified by the users. For example, it is possible to add some PTMs to those already present. The additional ASCII files are the following ones:

• *table_mw.txt *– It contains the list of the average and monoisotopic weights of the amino acids deprived of a molecule of water (see Table 1);

• *table_pi.txt *– It contains the amino acids involved in the computation of pI, their charge polarities and pKr values (see Table 2 Lehninger values);

• *table_ptm.txt *– It contains the names of all the considered PTMs, the involved amino acids, the MWs (monoisotopic and average) and the typology of PTMs (fixed or variable);

• *organism.txt *– It contains the complete list of the organisms for which a reference database can be created.

Moreover, in the *src *folder there is a setting file (*settings.ini*) that contains some parameters necessary to run the MsPI scripts (see the README file).

The main routines of the MsPI Perl tool are the following: *swiss2MsPI.pl*, *create_database.pl*, *create_database_random.pl*, *score_PMF.pl *and *make_grid.pl*. These routines run from the command line and interact with the user in the command line environment asking some parameters and options. They are briefly described in the following paragraphs.

#### Swiss2MsPI.pl

The *swiss2MsPI.pl *script uses the *lwp-download *program to download the database from a specific ftp address and saves the downloaded compressed file in the *tmp *directory. In the current version of the tool, the routine downloads the last version of the Swiss-Prot database. The ftp address is read from the *settings.ini *file and the database downloaded is renamed as swissprot_rel.xxx.fasta.gz, where *xxx *indicates the Swiss-Prot release.

#### Create_database.pl

The *create_database.pl *routine reorganizes the downloaded database and creates the reference database as illustrated in the Methods section. The user-dependent parameters necessary to create the new database are read from the *settings.ini *file. This routine includes also two subroutines for the computation of protein MW and pI. It first reads from the *tmp *folder the downloaded protein database and since it is stored in a compressed file, the Perl package IO::Zlib is used to read it at the fly without a preliminary decompression. The organisms to be considered are read from the file *organism.txt *and consequently proteins are selected. MWs and pIs are computed and the *in silico *enzymatic digestion is performed considering also the MCs and the PTMs as specified in the *settings.ini *file. The last step of this routine is the creation of a file containing the contaminant masses that should be removed from the query mass list. The accession numbers of the contaminants to be considered are placed in the *settings.ini *file. During the reference database creation, the number of times that a same peptide mass occurs is counted through a hash table to compute its frequency afterwards. This information is used by the routine *score_PMF.pl *to decide if to remove or not the contaminant masses from the query mass list on the basis of a user-defined threshold.

#### Create_database_random.pl

The *create_database_random.pl *routine creates the random database for the statistical validation of the ranked protein list obtained by the identification procedure. On the basis of the number of proteins in the reference database, the routine suggests to the user the "optimal" size of the random database supporting the choice of the *D*_*rnd *_parameter.

#### Score_PMF.pl and make_grid.pl

The *score_PMF.pl *script reproduces the third step of PMF, i.e. the search of the acquired spectrum in the reference protein database. The Perl packages used by this script are IO::Zlib, Math::BigInt and Math::BigFloat. The two latter allow to limit overflow and underflow problems. They are used in the computation of the similarity score when Binomial coefficients are too large.

First, the m/z contained in the input peak list are transformed into masses to be comparable with those of the peptides in the reference database and then, if the analyst wants to remove contaminants, the algorithm proceeds with the removal step. The list of the contaminant proteins is stored in the *contaminants.txt *file. The script selects which masses to remove from the query mass list on the basis of the protein accession number and of the peptide mass frequency in the reference database. Note that if a query mass matches that of a contaminant, it is removed from the query mass list and consequently the list shortens affecting the *L *parameter of the scores (see the Methods section).

If the mass range chosen by the user for a specific analysis is not equal to that stored in the *settings.ini *file and used to build the reference database, the identification takes longer because the number of peptides of each protein in the new mass range must be re-evaluated. Conversely, the routine directly reads this information in a suitable file created during the reference database building step.

In the searching step, the routine compares the query masses with the theoretical peptides which have an ID that satisfies the PTMs and MCs conditions imposed by the user, opening the right database files. When a match occurs, a hash table storing the total number of matches for that protein is updated. Moreover, further hash tables are created for computing the protein sequence coverage and for updating the protein MW on the basis of the MCs and PTMs that occur in its peptides. When all the database files are examined, MW and pI information on the unknown protein are used, if provided, to select the candidate proteins to be scored. They can be derived, for example, from the electrophoresis experiment. The tolerance window for MW is fixed to ± 20% while for pI it is fixed to ± 1. Both tolerances can be modified by the user.

On the basis of the scoring method chosen by the user (1, 2, or 3) a different algorithm is used to score each selected protein. In particular, in score 1 and 3 the Binomial coefficient is computed using a specific subroutine and if required and possible the Binomial distribution is approximated with a Gaussian distribution using a subroutine that evaluates the complementary error function as described in [27] and reported here in the next paragraph. If score 3 is chosen, the peptide mass distribution in the reference database is estimated if it is not assumed to be uniform. To this aim the routine uses the script *make_grid.pl *which creates the discretization grid described in the Methods section. If a grid file already exists in the database subfolder, the routine *score_PMF.pl *directly reads it and computes the parameter *λ*. When all the proteins have a score, they are ranked according to this score and the ranked candidate protein list is generated. If required, the p-value is computed thanks to the random database. This list is written in an output file, whose name is chosen by the user, and stored in the *results *folder. This file contains for all the candidate proteins the accession number, the ID, the organism, a description, the MW, the pI, the total number of amino acids, the number of query masses, the number of matches, the score, the p-value (if computed), the quality index (if computed), the protein sequence coverage and the list of matched peptides.

### The Gaussian approximation

The computation of scores 1 and 3 required the evaluation of the Binomial coefficient that, in some cases, can become so big to cause overflow. This problem can be overcome implementing the Gaussian approximation of the Binomial distribution. A Binomial distribution can be well-approximated with a Gaussian distribution of mean *μ *= *Lp *and variance *σ*^2 ^= *Lp*(1 - *p*) (N.B. for the score 3, *p *= *λ*) if:



This approximation is correct from a statistical point of view, because for *L *tending to infinite, the Binomial distribution is asymptotically Gaussian.

The cumulative Gaussian distribution probability is:

(5)

Being to Binomial distribution a discrete distribution and the Gaussian distribution a continuous distribution, to approximate the Binomial distribution with the Gaussian distribution it is suitable to apply the so called Continuity Correction defining:

(6)

This correction is less accurate in the tails of the Gaussian distribution and it introduces some numerical errors in the evaluation of (6), being the Gaussian cumulative probability distribution not available in closed form by elementary functions. Nevertheless, the integral at the second member of the (5) can be computed by a numeric way through the error function *erf*(*x*):



There is a connection between the error function and the standard Gaussian cumulative distribution *P*(*X *<*x*), because they differ only for translation and scaling [28]:



The probability can be computed using the error function, but typically the *complementary error function *is used, because it is more accurate for *x *values greater than 0.5, especially in the Gaussian tails and, moreover, it is reported in the literature [29]:



From a computational point of view, the probability of a Gaussian random variable is calculated by the rational Chebyshev approximation for the *erfc*(*x*) [27]. The *erfc*(*x*) approximation is made considering three intervals and using rational polynomial on the basis of the interval where *x *falls. In particular:



where the random Gaussian variable *x *is standardized and the coefficients *p*_*j *_e *q*_*j *_are reported in the literature. In this work, the maximum polynomial degree was used.

## Competing interests

The authors declare that they have no competing interests.

## Authors' contributions

AT led the design and the implementation of the Perl procedure, the software tool comparisons and drafted the manuscript. NB helped to define the analysis procedure. ST and LR designed and performed the PMF experiments, led a first analysis of the data with commercial software tool and contributed to the definition of the overall procedure. PM supervised the design and the implementation of the Perl procedure and finalized the manuscript. All authors read and approved the final version of the manuscript.
